# Multifunctional GO-Based Hydrogels with Various Inorganic Additives for Oral Health and Photodynamic Activation

**DOI:** 10.3390/gels12010046

**Published:** 2026-01-01

**Authors:** Codruta Sarosi, Marioara Moldovan, Ioan Petean, Miuta Filip, Gabriel Furtos, Sonia Balint, Rahela Carpa, Andrei Cristian Ionescu

**Affiliations:** 1 Department of Polymer Composites, Institute of Chemistry Raluca Ripan, Babeș-Bolyai University, 30 Fantanele St., 400294 Cluj-Napoca, Romania; liana.sarosi@ubbcluj.ro (C.S.); gabriel.furtos@ubbcluj.ro (G.F.); sonia.balint@ubbcluj.ro (S.B.); 2 Faculty of Chemistry and Chemical Engineering, Babeș-Bolyai University, 11 Arany Janos St., 400028 Cluj-Napoca, Romania; ioan.petean@ubbcluj.ro; 3 Department of Analytical Chemistry, Institute of Chemistry Raluca Ripan, Babeș-Bolyai University, 30 Fantanele St., 400294 Cluj-Napoca, Romania; miuta.filip@ubbcluj.ro; 4 Department of Molecular Biology and Biotechnology, Faculty of Biology and Geology, Babeș-Bolyai University, 1 M. Kogalniceanu Street, 400084 Cluj-Napoca, Romania; rahela.carpa@ubbcluj.ro; 5 Oral Microbiology and Biomaterials Laboratory, Department of Biomedical, Surgical, and Dental Sciences, University of Milan, Via Pascal 36, 20133 Milan, Italy; andrei.ionescu@unimi.it; 6 Fondazione IRCCS Ca’ Granda Ospedale Maggiore Policlinico, 20122 Milan, Italy

**Keywords:** hydrogel, graphene oxide, photodynamic therapy (PDT), antibacterial, SEM

## Abstract

In this study, we present the synthesis and characterization of graphene oxide (GO)-based hydrogels reinforced with hydroxyapatite (HA), titanium dioxide (TiO_2_), zinc oxide (ZnO), silicon oxide (SiO_2_), silver (Ag), and graphitic carbon nitride (g-C_3_N_4_). The aim is to develop multifunctional hydrogels with enhanced structural and biological performance and photocatalytic activity, opening the way for applications in regenerative medicine. The structure and composition of the hydrogels were investigated using FTIR and UV–Vis spectroscopy, which highlighted the chemical interactions between GO and the incorporated nanoparticles. The morphology was analyzed through scanning electron microscopy (SEM) and metallographic optical microscopy (MOM), confirming a uniform distribution of the inorganic phases and an internal architecture optimized for stability and bioactivity. Antibacterial activity was evaluated against Gram-positive and Gram-negative strains, both in the absence and presence of photodynamic therapy. The latter was activated by a Woodpecker laser at a 420 nm wavelength. The results showed significant bacterial inhibition, further enhanced by laser exposure, suggesting a synergistic effect between photocatalytic activation and the hydrogel components. Overall, the obtained hydrogels demonstrate robust mechano-structural properties and promising biological activity, supporting their potential for innovative biomedical applications in the tissue regeneration field and for the emerging biofunctional technologies.

## 1. Introduction

Hydrogels are three-dimensional (3D) networks made of hydrophilic polymer chains [1,2], recognized for their strong water affinity, high swelling capacity, micro-nanoscale porosity, and mechanical softness. These materials can absorb significant quantities of water, often exceeding 90% of their total weight, while remaining insoluble in aqueous environments [3]. Their structural stability in hydrated conditions and the consequent behavior arise from physical or chemical crosslinking among the hydrophilic polymer chains [4]. Their hydrated stability and porous architecture enable the diffusion of small molecules, while their tissue-like behavior allows them to mimic soft biological environments, making hydrogels attractive for biomedical applications such as controlled drug delivery, tissue engineering scaffolds, and wound healing [5,6,7,8,9,10].

Hydrogels are commonly synthesized using natural and synthetic polymers. Among the most frequently employed natural polymers are hyaluronic acid, alginate, chitosan, collagen, and gelatin, while synthetic options include polyethylene glycol (PEG), poly(acrylamide) (PAAm), poly(2-hydroxyethyl methacrylate), and poly (acrylic acid) (PAA) [11,12]. By selecting the polymer type, crosslinking agents, and crosslinking density, hydrogel properties such as water content, permeability, and responsiveness to external stimuli can be adjusted for specific biomedical applications [13]. Owing to this versatility, hydrogels can protect encapsulated drugs from harsh physiological environments, such as the oral one, and modulate their release in response to external stimuli as temperature, pH, or light [14,15,16]. Hydrophilic and hemostatic hydrogels use their water-rich, lubricated surfaces and multiple weak/strong molecular interactions to prevent protein adhesion, resist friction, and securely adhere to tissues while promoting rapid wound sealing and healing [17]. Despite their promising features, traditional hydrogels often face structural limitations that can restrict their therapeutic applicability. These materials frequently exhibit vulnerabilities under external forces, which may compromise their integrity during handling or application. This highlights the need for reinforced or molecularly engineered gel systems capable of maintaining stability and functional performance under various conditions [18]. To address this limitation, the incorporation of nanoadditives has emerged as an effective approach to reinforce their networks and expand their functionality. Graphene and its derivatives have garnered considerable attention among the diverse nanomaterials studied, owing to their distinctive physicochemical and biological characteristics. To address these limitations of conventional polymer matrices, nanocomposite approaches have been widely explored. The incorporation of nanoadditives can enhance network integrity, functional performance, and biological activity. Among various nanomaterials, graphene and its derivatives—particularly graphene oxide (GO)—have attracted significant attention due to their large surface area and rich surface chemistry [19,20,21,22]. GO contains abundant oxygen-containing functional groups (hydroxyl, epoxy, and carboxyl), which enable strong hydrogen bonding and electrostatic interactions with polymer chains, contributing to improved network cohesion and multifunctionality [23]. Single-component natural hydrogels typically exhibit inadequate mechanical strength and stability, limiting their standalone use. Consequently, their performance is often enhanced through chemical modification or by integrating them with additional materials [24].

The incorporation of HA, TiO_2_, ZnO, SiO_2_, Ag, and g-C_3_N_4_ nanoparticles into various hydrogel systems, as well as their use in photodynamic therapy (PDT) for cancer treatment and oral health applications, has been previously explored. However, hydrogel-based films combining graphene oxide (GO) with metallic or metal-oxide nanoparticles for treating oral soft-tissue disorders have not yet been reported [25,26]. Graphene oxide, with its large surface area, abundant oxygen-containing functional groups, and excellent mechanical and electrical properties, serves as an effective reinforcing agent and provides active sites for nanoparticle anchoring. When combined with metal and metal-oxide nanoparticles such as TiO_2_, ZnO, SiO_2_, or Ag, the resulting composites can exhibit multifunctional properties, including antimicrobial activity, enhanced photo-catalytic behavior, and improved structural stability [27]. The addition of hydroxyapatite (HA), a bioceramic closely resembling the mineral phase of bone, further increases bioactivity and promotes cell attachment, making these hybrid hydrogels promising for biomedical and tissue-engineering applications. g-C_3_N_4_ also demonstrates strong antimicrobial activity, attributed to its ability to disrupt bacterial membrane integrity and generate reactive oxygen species (ROS) [28]. Given that biofilms are implicated in an estimated 80% of microbial infections, materials capable of inhibiting biofilm formation are relevant, particularly in the context of oral health. While hydrogels offer clinical advantages such as maintaining a moist environment, absorbing exudates, self-healing to preserve structural stability, and strong adhesion to wound sites, advances in 2D materials like graphene further expand the potential for next-generation wound dressings [29]. The incorporation of such graphene-based nanomaterials into hydrogel matrices not only enhances their mechanical strength and elasticity but also introduces electrical conductivity and photoresponsiveness, properties that are critical for light-activated therapeutic applications. These hybrid systems have been increasingly explored as smart materials for phototherapy, particularly in photodynamic therapy (PDT).

Photodynamic therapy is a minimally invasive treatment modality that employs light, photosensitizers (PSs), and oxygen to generate reactive oxygen species (ROS), which induce localized cytotoxicity in target cells or microorganisms. PDT has found broad ap-plications in oncology, dermatology, dentistry, and antimicrobial therapy due to its precise spatial control and low systemic toxicity for the treatment of cancer, bacterial infections, and tissue repair and regeneration [30,31]. Photodynamic therapy uses near-infrared-activated photosensitizers to selectively destroy cancer cells, offering a precise, minimally invasive treatment that spares healthy tissue [32]. However, the effectiveness of PDT relies heavily on the efficient delivery and retention of photosensitizers at the target site, as well as on maintaining their photostability and biocompatibility in biological environments [33,34].

Hydrogels offer an ideal platform for PDT because of their high water content, biocompatibility, and ability to encapsulate and release photosensitizing agents in a controlled manner [35,36]. The combination of hydrogels with graphene-based nanomaterials provides additional advantages: graphene’s extended π-conjugated structure allows efficient energy transfer and improved light absorption, enhancing ROS generation under irradiation. Furthermore, graphene and its derivatives can serve as carriers or stabilizers for photosensitizers, facilitating their uniform dispersion within the hydrogel and improving their photodynamic efficiency [30,37,38,39].

This work introduces a multifunctional GO-based hydrogel reinforced with multiple inorganic additives—hydroxyapatite, TiO_2_, ZnO, SiO_2_, Ag, and g-C_3_N_4_—using a single matrix. The novelty of this study lies in the combination of these fillers, including the photocatalytic g-C_3_N_4_, to create a unique nanocomposite with synergistic structural and biological functionalities, offering new opportunities for oral health applications and photodynamic therapies.

In this study, we report the synthesis and comprehensive characterization (SEM, MOM, FT-IR, UV–Vis, and antibacterial assays) of these GO-based hydrogels. The objective was to develop a multifunctional hydrogel with enhanced structural integrity and antibacterial activity, leveraging photodynamic therapy, and highlighting its potential for oral health applications.

## 2. Results and Discussion

Eight types of hydrogels were made (Table 1) and noted as follows: H1: Graphene oxide (GO); H2: Graphene oxide with hydroxyapatite; H3: Graphene oxide with zinc oxide (GO-ZnO); H4: Graphene oxide with titanium dioxide (GO-TiO_2_); H5: Graphene oxide with silica oxide (GO-SiO_2_); H6: Graphene oxide with silver (GO-Ag); H7: Graphitic carbon nitride (g-C_3_N_4_), and H0: Control hydrogel without nanoparticles.

The characterization included compositional analysis by FTIR and UV–Vis spectroscopy, and morphological analysis by mineralogical optical microscopy and scanning electron microscopy. The antimicrobial testing concluded the analyses, as detailed below.

### 2.1. FTIR Spectra

**Figure 1:**
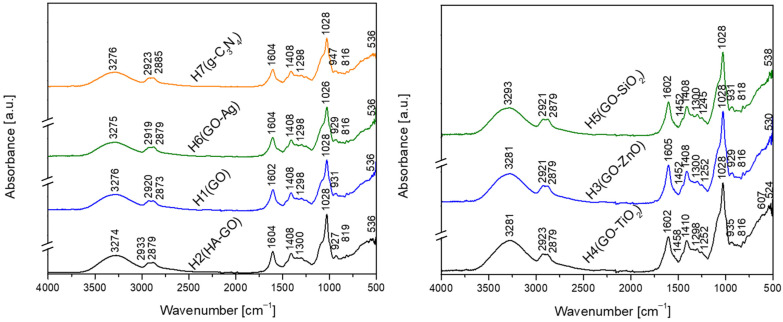
The FTIR spectra of the tested hydrogel films (**H1**–**H7**).

All FTIR spectra of the tested hydrogels show a broad absorption band between 3000 and 3500 cm^−1^, attributed to N–H stretching, the O–H stretching vibrations of alginate, and the residual water molecules within the network [30,38,39]. Aliphatic C–H stretching vibrations are observed at 2923–2879 cm^−1^. The bands at 1602 and 1452 cm^−1^ correspond to the asymmetric and symmetric stretching vibrations of carboxylate salt ions, respectively [40], while the asymmetric stretching of carboxylate groups appears at 1635 and 1604 cm^−1^ [41]. The peaks at 1298 and 1252 cm^−1^ are assigned to the stretching vibrations of C–N (−C)–C or C–NH–C bonds [30] (Figure 1).

Overall, in the GO–ZnO, GO–TiO_2_, and GO–SiO_2_ composites, metal–oxygen (M–O) vibrations are observed in the 400–700 cm^−1^ region. Concurrently, the partial disappearance of the C=O and C–O bands confirms the coordination of metal species with the oxygen functionalities of GO, indicating strong interfacial interactions and successful integration of inorganic fillers within the hydrogel network.

In the GO–Ag nanomaterial, a peak at 929 cm^−1^ indicates the interactions between Ag nanoparticles and the oxygenated functional groups of GO. The GO–HA spectrum exhibits characteristic O–P–O vibrations at 536 and 602 cm^−1^, along with a strong PO_4_^3−^ band in the range of 1030–1090 cm^−1^. For the GO–TiO_2_, the band at 1020–1070 cm^−1^ is attributed to Ti–O–C linkages, confirming the presence of a chemical bonding between TiO_2_ and the oxygen functionalities of GO. In the GO–ZnO composite, a peak at 550–600 cm^−1^ is attributed to Zn–O and is accompanied by features at 1040–931 cm^−1^ corresponding to C–O and Zn–O–C interactions. The GO–SiO_2_ spectrum shows absorption bands at 1090–1070 cm^−1^ (Si–O–Si) and 818 cm^−1^ (Si–O).

The FTIR spectra of the GO-based hydrogels show both general patterns and interactions between the fillers and the GO matrix. Distinct filler signatures are observed in each hydrogel. The presence of PEG 400 and glycerine in the experimental hydrogels, used as plasticizers, promotes the mobility of the polymer chains and contributes to an increased swelling degree. Moreover, the addition of graphene-based nanoparticles results in a slight reduction in the swelling degree compared to systems without nanofillers, suggesting the formation of additional interactions, such as hydrogen bonding and electrostatic interactions, between the functional groups of the alginate, whey proteins, and the surface of graphene oxide. These interactions act as points of physical pseudo-crosslinking, leading to a more compact network structure and improved structural stability of the hydrogel, without involving true chemical or ionic crosslinking. This evidence confirms that the inorganic fillers are not merely physically embedded but also chemically interact with the GO sheets, contributing to the structural integrity and functional properties of the hydrogel network.

### 2.2. UV–Vis Spectra

**Figure 2:**
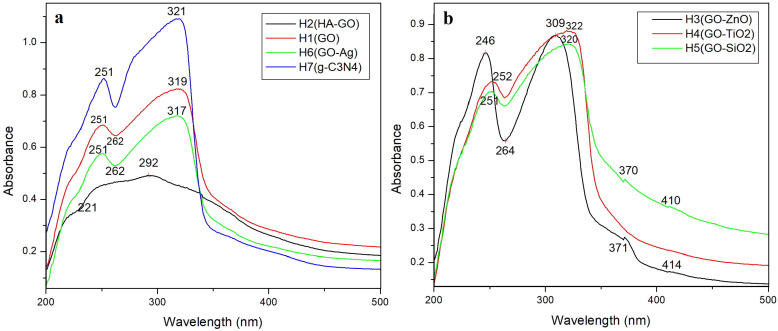
UV–Vis spectra of GO, HA-GO, GO-Ag, and g-C_3_N_4_ hydrogels (**a**); UV–Vis spectra of GO-ZnO, GO-TiO_2_, and GO-SiO_2_ hydrogels (**b**).

The UV–Vis spectra (Figure 2a) were processed by subtracting the spectrum of the blank hydrogel matrix (H0) from the spectra of the active formulations (H1–H7). This procedure was applied in order to isolate the absorbance contributions of the active components and to eliminate the background originating from the hydrogel matrix itself (glycerol, PEG 400, and sodium alginate). The subtraction highlights the intrinsic optical features of GO, HA–GO, GO–Ag, and g-C_3_N_4_, enabling a more accurate comparison between samples [27,42].

The UV–Vis spectra of the investigated hydrogels reveal significant changes in the electronic transitions of graphene oxide (GO) depending on the introduced component. The absorbance band at ~262 nm is not associated with C=C double bonds in alginate or with alginate degradation, but rather with GO-related transitions.

The GO sample presents characteristic maxima at ~251 nm (π–π* transition) and ~319 nm (n–π* transition), specific to the conjugated aromatic domains and oxygenated functional groups. In the HA–GO hydrogel, the absorption is shifted to ~221 nm, and the overall band intensity decreases, indicating a reduction of π-conjugation due to interactions between HA and the oxygen-containing functionalities of GO. In the case of GO–Ag, the π–π* band remains around 251 nm, yet the modification of the spectral profile in the 300–330 nm region suggests an electronic influence of Ag nanoparticles, possibly related to a weak plasmonic contribution superimposed on the intrinsic GO transitions. The g-C_3_N_4_ sample exhibits the strongest absorption, with a pronounced band at ~321 nm, characteristic of n–π* transitions in tri-azine structures, confirming an extended electronic conjugation.

The UV–Vis spectra of the GO–ZnO, GO–TiO_2_, and GO–SiO_2_ hydrogels (Figure 2b) highlight the different ways in which metal oxides affect the optical transitions of graphene oxide. The GO–ZnO and GO–TiO_2_ samples show an enhancement of the π–π* band in the 246–252 nm region and a broad absorption band at ~309–322 nm, assigned both to the n–π* transitions of GO and to the characteristic absorption of semiconductor oxides. The broadening of the absorption tail in the 370–414 nm region indicates the formation of GO–ZnO and GO–TiO_2_ heterojunctions, which promote electron transfer and extend the absorbance into the visible domain. In contrast, the GO–SiO_2_ sample exhibits a spectral profile similar to that of neat GO, confirming the optically inert behavior of SiO_2_ and its minimal interaction with the electronic structure of GO. Altogether, these spectra demonstrate that each component (HA, Ag, and g-C_3_N_4_) modifies the electronic structure of GO in a distinct manner, influencing both the position and the intensity of the optical transitions [43]. Furthermore, these results demonstrate that ZnO and TiO_2_ significantly influence the photonic behavior of GO, while SiO_2_ predominantly acts as a dielectric support [44]. In this way, a method can be provided to tailor the optical transitions and therefore the photonic behavior of the GO depending on the required properties of the nanomaterial itself.

### 2.3. Mineralogical Optical Microscopy (MOM)

MOM investigation uses the cross-polarized light for revealing the crystalline structures within the tested hydrogels. Each crystal has its specific color due to its crystal lattice interference with the polarized light.

**Figure 3:**
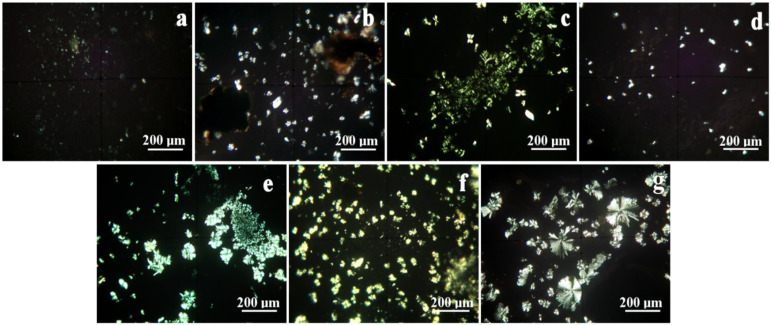
Mineralogical optical microscopy (MOM) images of the investigated hydrogels, containing: (**a**) H1: GO, (**b**) H2: HA-GO, (**c**) H3: GO-ZnO, (**d**) H4: GO-TiO_2_, (**e**) H5: GO-SiO_2_, (**f**) H6: GO-Ag, and (**g**) H7: g-C_3_N_4_.

The GO–ZnO hydrogel (Figure 3c) presents a heterogeneous morphology composed of small hexagonal particles (1–5 µm) intermixed with hexagonal acicular structures having diameters of approximately 5 µm and lengths of 10–20 µm. These features exhibit a lemon-yellow coloration. In contrast, the GO–TiO_2_ hydrogel displays a sparse and randomly distributed filler phase within the thin hydrogel film. The TiO_2_ aggregates form irregular, boulder-like structures with diameters between 3 and 25 µm, visible as white-toned regions (Figure 3d).

The GO–SiO_2_ hydrogel (Figure 3e) reveals uniformly dispersed SiO_2_ clusters with a greenish hue. These aggregates exhibit polyhedral to boulder-like morphologies ranging from 15 to 300 µm in size. Additionally, smaller, well-individualized quartz particles (<1 µm) are observed adjacent to the larger clusters. In the GO–Ag hydrogel (Figure 3f), the dispersion of the oxide phase is efficient, as indicated by the bright yellow coloration of the mineral filler contrasting with the dark brown GO sheets, which remain in the background.

Graphitic carbon nitride (g-C_3_N_4_) retains the sheet-like morphology characteristic of its graphitic precursor and shows homogeneous dispersion within the hydrogel matrix. The presence of nitrogen atoms bonded within the carbon framework induces a specific degree of crystallinity. Consequently, the micro-optical microscopy (MOM) image (Figure 3g) displays a radiant pattern of polarized light, reflecting the radial organization of g-C_3_N_4_ sheets. Their dimensions range from approximately 30 µm to large formations up to 180 µm.

Overall, this analysis reveals the uniform distribution of the mineral filler particles and their subsequent interlocking with the graphene oxide sheets. The peculiar disposition observed in ZnO, TiO_2_, SiO_2_, and Ag within the hydrogel matrix may facilitate the compounds’ contact with the membrane or cell wall of microorganisms, thus potentially evoking the antimicrobial features that are analyzed below. For the same reasons, the radial termination of the filler particles in the g-C_3_N_4_ samples may provide the basis for a supposed bactericidal activity upon contact, explored below. Wang et al. highlight trends in the development of stimulus-responsive nanocomposite hydrogels for wound healing and antibacterial applications, in which MXene-rGO and GO were incorporated [28].

### 2.4. SEM Microscopy

**Figure 4:**
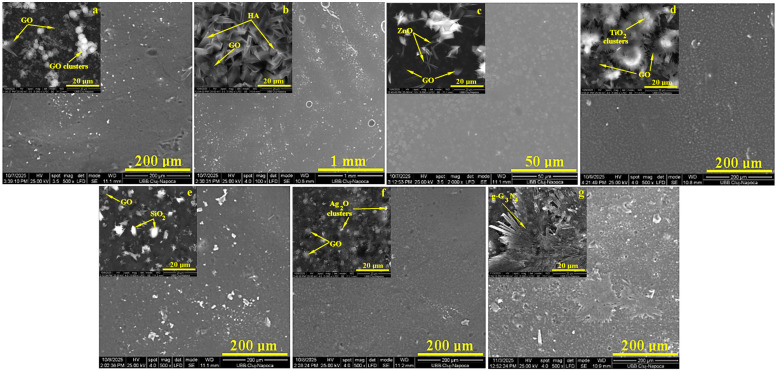
SEM images of the investigated hydrogels at a magnification of ×100–×500, containing: (**a**) H1: GO, (**b**) H2: GO-HA, (**c**) H3: GO-ZnO, (**d**) H4: GO-TiO_2_, (**e**) H5: GO-SiO_2_, (**f**) H6: GO-Ag, and (**g**) H7: g-C_3_N_4_; in the top left images, the indicated hydrogels are shown at a magnification of ×5000.

SEM images of the investigated hydrogels containing different GO-based and g-C_3_N_4_ nanofillers are presented in Figure 4. At low magnification (×100–×500), all hydrogels exhibit a continuous and interconnected polymeric network with a relatively rough and heterogeneous surface morphology, which is typical for dried hydrogel systems. No large cracks or macroscopic phase separation are observed, indicating good structural integrity and compatibility between the polymer matrix and the nanofillers.

The SEM micrographs in Figure 4a (top) reveal a homogeneous and compact hydrogel network in which the filler particles are uniformly embedded within the polymeric matrix. The graphene oxide (GO) sheets exhibit predominantly rectangular morphologies with lateral dimensions ranging from 3 to 20 µm.

In the GO–hydroxyapatite (GO–HA) hydrogel (Figure 4b—top), the HA phase is represented by well-defined needle-like crystals with an average length of approximately 15 µm and a diameter near 5 µm. These elongated structures are homogeneously distributed throughout the matrix and oriented in relation to the GO sheets, contributing to the formation of a robust and structurally coherent composite network.

The GO–ZnO hydrogel (Figure 4c—top) displays acicular ZnO structures with lengths around 10 µm and widths below 3 µm. The ZnO needles exhibit preferential association with GO sheets, forming intricate hybrid clusters that enhance the structural connectivity and functional integration within the hydrogel.

In the GO–TiO_2_ and GO–SiO_2_ systems (Figure 4d,e—top), both fillers are well encapsulated within the hydrogel framework, indicating strong interfacial interactions and uniform microstructural stability. These oxides appear to reinforce the polymer network by limiting phase segregation and maintaining consistent filler distribution across the matrix.

The GO–Ag hydrogel (Figure 4f—top) presents a refined and more compact gel structure, where Ag nanoparticles are well dispersed throughout the matrix. The presence of these fine micro-fillers enhances the homogeneity of the composite and likely improves the overall functional properties of the hydrogel.

The SEM micrograph of g-C_3_N_4_ (Figure 4g—top) reveals a well-developed microstructure consisting of a dense central core, derived from the graphite precursor, and radially oriented prismatic whiskers forming a compact sheet-like arrangement. The outer whisker termini appear partially fragmented due to interactions and exfoliation during formation. These detached fragments are evenly distributed within the hydrogel as discrete filler particles with sizes ranging from 2 to 20 µm, which correlates well with the micro-optical microscopy (MOM) findings.

The morphological evidence suggests that the close relationship between GO sheets and the inorganic fillers controls how well the hybrid hydrogels work as a whole. The uniform distribution of the fillers and their strong interfacial contact with the GO matrix are expected to enhance mechanical reinforcement through effective stress transfer and network densification. In particular, the acicular morphologies of HA and ZnO contribute to improved rigidity and load-bearing capacity, while the TiO_2_ and SiO_2_ phases stabilize the gel microstructure by minimizing local deformation and shrinkage during drying. The fine dispersion of Ag nanoparticles enhances the homogeneity and may impart antimicrobial activity, whereas the lamellar and radially organized g-C_3_N_4_ domains promote electrical and photocatalytic functionalities. Therefore, the correlated MOM and SEM analyses confirm that the designed hybrid architecture successfully integrates structural uniformity with tailored properties, making these GO-based hydrogels promising materials for biomedical [29], catalytic, and environmental applications. At higher magnification (×5000), the specific sites involved microstructurally between filler particles and hydrogel matrix are observed. This observation is expected considering the low nanofiller content and the fact that the nanoparticles are embedded and partially covered by the polymer matrix. Instead of discrete particles, the nanofillers manifest as subtle variations in surface roughness and local densification, confirming their successful incorporation into the hydrogel rather than surface accumulation or phase separation.

Overall, the SEM observations confirm the formation of well-integrated nanocomposite hydrogels, in which the different nanofillers are effectively dispersed within the polymer matrix and contribute to the modulation of surface morphology without compromising the structural continuity of the hydrogel.

### 2.5. Antibacterial Activity of the Hydrogel Samples

**Figure 5:**
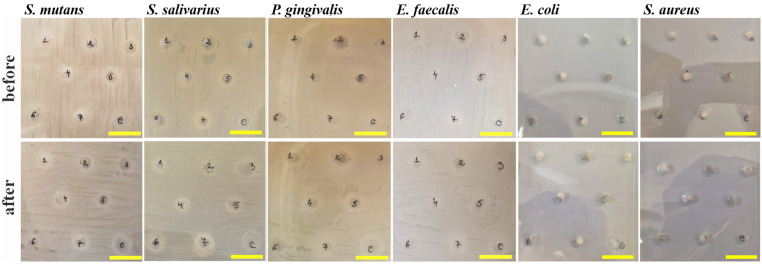
Antibacterial inhibition zones measured for the experimental hydrogels tested on pathogens before and after laser irradiation. The number corresponds to the tested hydrogel formulation, as follows (1) GO, (2) HA-GO, (3) GO-ZnO, (4) GO-TiO_2_, (5) GO-SiO_2_, (6) GO-Ag, and (7) g-C_3_N_4_. C represents the control sample that contains no nanofiller particles. The yellow scale bar has a length of 20 mm.

Literature reveals that the laser irradiation of dental composites might increase their antibacterial activity due to the increased embedding of bioactive compounds related to the filler particles [45,46]. As a localized treatment, photodynamic therapy offers minimal invasiveness, low risk of drug resistance, real-time diagnostic capability, and strong tissue selectivity, allowing photosensitive materials to accumulate at the illuminated site without harming nearby healthy tissues [32]. Thus, the irradiation effect on the antibacterial activity was tested on our hydrogels. Figure 5 reveals the inhibition zones for the tested hydrogels before and after laser irradiation.

Two steps of statistical analysis were performed regarding the obtained values. The first step checked the antibacterial activity before and after irradiation, the significance value a = 0.05, and the samples having *p* > a present statistical similarity (e.g., constant values or minor changes like slight increases or decreases regarding the mean control value).

**Figure 6:**
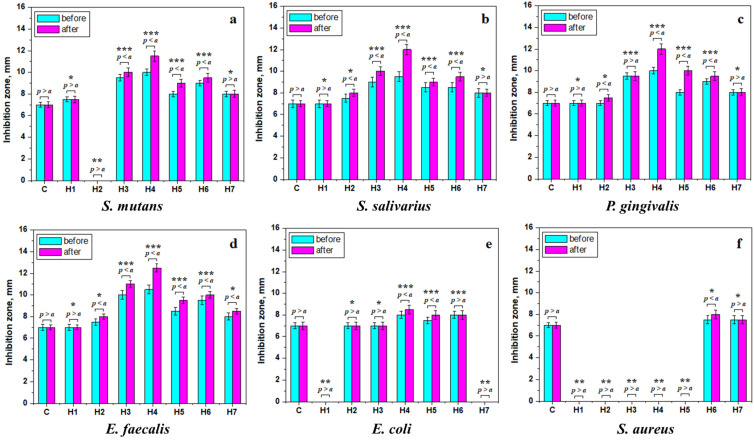
Antibacterial activity as mean inhibition zone for the tested hydrogels before (light-blue) and after (magenta) laser irradiation against the following pathogens: (**a**) *Streptococcus mutans*, (**b**) *Streptococcus salivarius*, (**c**) *Porphyromonas gingivalis*, (**d**) *Enterococcus faecalis*, (**e**) *Escherichia coli*, and (**f**) *Staphylococcus aureus*. Statistically significant differences of the same material before and after irradiation are indicated by the *p*-variance compared to a significance level of a = 0.05, while groups with the same statistical significance are marked with the same number of asterisks. * The first group includes inhibition zones that are similar to the control sample, with only minor increases or decreases. ** The second group has a significant decrease in the inhibition zone regarding the control value (e.g., some of the samples reveal no inhibition). *** The third statistical group contains samples having a strong increase in the inhibition zone regarding the control samples.

The second step of statistical analysis is based on the inhibition zone thresholds given by the control sample, which exhibited a mean inhibition zone of 7 mm. Three statistical groups were identified, marked with asterisks in Figure 6.

The literature data demonstrate that *Streptococcus mutans* is often found in the oral microbiota and is associated with an increased caries risk, making it a very good marker for the disease [47]. Therefore, it is critical to observe its interaction with our hydrogels. We observe in Figure 6a that H1 and H7 feature a similar inhibition zone to the control, thus belonging to the first statistical group. Sample H2 has no inhibition, but the greatest differences are displayed by the H3, H4, H5, and H6 hydrogels, revealing an increased inhibition compared to the control sample and after laser irradiation.

*Streptococcus salivarius* is another component of the oral microbiota that is not pathogenic per se. Then again, in certain cases, in weak and immunocompromised patients, it might cause complications, such as meningitis [48]. Figure 6b reveals that H1, H2, and H7 are situated in the first statistical group, having an inhibition zone similar to the control sample, and a slight increase in the inhibition zone after irradiation. Interestingly, samples H3, H4, H5, and H6 present a significant increase in the inhibition zone compared to the control, and as a consequence of the irradiation. This datum indicates a synergistic effect between the hydrogel matrix and the filler components of the latter materials.

Figure 6c depicts the antibacterial effect against *Porphyromonas gingivalis*. This microorganism is a well-known member of the oral microbiota associated with gingivitis and periodontitis [49,50]. Recent studies also reported it to play a significant indirect role as a determinant for Alzheimer’s disease [49], and it might also be implicated in the promotion of oral cancer by modulating molecular and genetic factors [28,51]. The inhibition of this pathogen is therefore paramount for preserving oral health. Figure 6c shows an overall good antimicrobial behavior. More specifically, H1, H2, and H7 have similar behavior to the control sample, being situated in the first statistical group. The filler addition and irradiation have less effect on the hydrogel antibacterial activity. The latter is strongly increased by H3, H4, H5, and H6, which evidence increased inhibition zones compared to the control and after irradiation. A similar antimicrobial behavior was observed against *Enterococcus faecalis*, Figure 6d, revealing the same activity by the tested hydrogels. *E. faecalis* is specific for the gut microbiota, even being a probiotic [52], but it is rather dangerous when it occurs in the oral environment, as it is involved in periodontal disease and is responsible for many infections of the dental pulp [53,54]. Thus, the samples H3, H4, H5, and H6 are very effective against E. faecalis, while H1, H2, and H7 have a moderate effect, induced more by the polymeric matrix of hydrogel and less by the filler particles.

*Escherichia coli* is a relatively dangerous and potentially deadly pathogen when ingested [55,56,57]. The tested hydrogels have a relatively modest antimicrobial effect on this pathogen, as shown in Figure 6e. Samples H2 and H3 belong to the first statistical group, having a similar inhibition zone to the control sample, while H1 and H7 have no inhibition, thus corresponding to the second statistical group. On the other hand, samples H4, H5, and H6 have a relevant increase in the inhibition zone compared to the control sample, indicating a positive antibacterial effect of their filler particles [58,59].

*Staphylococcus aureus* is a very dangerous pathogen implicated in nosocomial infections and presenting a high risk of death for patients having other affections [57,60]. Figure 6f shows evidence that the control sample has a steady behavior regardless of irradiation exposure, revealing no significant statistical difference in contact with this pathogen; both situations have an inhibition zone of 7mm. Hydrogels H6 and H7 have an antimicrobial behavior similar to the control sample after irradiation, belonging to the first statistical group, while the other samples have no inhibition [61].

Overall, the results show that the best antimicrobial effect is exhibited by the following filler particles: GO-TiO_2_, GO-ZnO, GO-Ag, and GO-SiO_2_. The moderate antimicrobial effect was induced by the following filler particles: GO–HA, GO–freeze-dried, and g-C_3_N_4_.

A significant challenge for phototherapeutic hydrogels remains their mechanical fragility, which can limit their use in tissue-contact applications [62]. The incorporation of graphene-based nanostructures effectively addresses this limitation by reinforcing the polymeric network and improving its resemblance to the natural extracellular matrix [63]. This makes them promising candidates for biomedical, photocatalytic, and oral health applications where multifunctionality and light-triggered antimicrobial activity are desirable.

## 3. Conclusions

The characterization of GO-based hydrogels demonstrates that the inorganic fillers are not merely physically embedded but also chemically interact with the GO matrix. FTIR spectra revealed both common GO features and distinct filler-specific signatures, confirming the formation of strong GO–filler interactions that contribute to the structural integrity and functional properties of the hydrogels. UV–Vis analyses further supported these interactions, indicating modifications in electronic transitions consistent with hybrid formation. Morphological observations from SEM and MOM confirmed a uniform and intimate association between GO sheets and inorganic fillers, highlighting that the hybrid architecture effectively integrates structural uniformity with multifunctional properties.

Antibacterial activity varied among species, with Streptococci, *P. gingivalis*, and *E. faecalis* being most susceptible to metal-doped GO hydrogels, particularly H6. Only H6 and H4 showed significant activity against *E. coli* and *S. aureus*. Photodynamic therapy (PDT) significantly enhanced antibacterial performance, especially when hydrogels contained photocatalytic components such as g-C_3_N_4_.

Overall, these results indicate that the designed GO–filler hybrid hydrogels combine chemical, structural, and morphological advantages with potent antibacterial functionality, especially when exposed to irradiation. Hydrogels offer an effective antibacterial platform by pairing a form of antibiotics with a highly reactive, high-surface-area carrier that enhances drug release and bacterial elimination.

## 4. Materials and Methods

### 4.1. Preparation of Hydrogels

Seven hydrogel formulations were prepared using identical amounts of glycerine, sodium alginate solution (1:0.5 *w*/*w*), and whey protein isolate (WPI) solution at a concentration of 2% (*w*/*w*) (all from Remed Prodimpex SRL, Bucuresti, Romania). The components were mixed for 2 h at 800 rpm using an AREX heating magnetic stirrer (VELP Scientifica, Usmate, Italy) to ensure complete dissolution and homogeneity. Separately, 2 mL of polyethylene glycol (PEG400, from Sigma-Aldrich, Darmstadt, Germany) was homogenized with graphene powder, and the resulting dispersion was subsequently incorporated into the hydrogel matrix. After homogenization, salicylate (0.015%; Merck Schuchardt OHG, Hohe Brunn, Germany) was added to improve stability and prevent degradation over time.

All powders were synthesized in the UBB-ICCRR laboratory (Institute of Chemistry Raluca Ripan, Babeș-Bolyai University, Cluj-Napoca, Romania), according to previously reported protocols [64,65,66]. Graphene oxide was synthesized using the Hummers method, and the reaction product was lyophilized to obtain nanoscale GO particles [67]. The compositions of the experimental hydrogel formulations (H1–H7) are reported in Table 1. A control hydrogel without powder addition (H0) was also formulated.

### 4.2. FTIR and UV–VIS Spectroscopy

The hydrogels were analyzed using Fourier-transform infrared spectroscopy (FTIR) on a JASCO FTIR 610 spectrometer (JASCO Corporation, Tokyo, Japan). Measurements were carried out in the 4000–400 cm^−1^ wavenumber range employing the ATR (attenuated total reflectance) mode. Spectra were collected at a resolution of 4 cm^−1^, with 100 scans performed for each acquisition.

Absorption spectra were acquired at room temperature using a JASCO V-750 UV–Visible spectrophotometer (JASCO Corporation), equipped with a double-beam optical system and automatic radiation-source switching. Measurements were performed in absorbance mode over the 900–200 nm range, with a 5 nm slit width and a 1 nm data pitch.

### 4.3. Mineralogical Optical Microscopy (MOM)

Microscopy observations were performed with a Laboval 2 Pol microscope under cross-polarized light (Zeiss Company, Obercochen, Germany), equipped with a digital image-acquiring system (Samsung 10 MPx, Samsung Electronics Co., Ltd., Suwon, Republic of Korea).

### 4.4. SEM Microscopy

SEM analysis of the hydrogels was performed using an Inspect scanning electron microscope (FEI Company, Hillsboro, OR, USA) operated in low-vacuum mode at an accelerating voltage of 20 kV. The samples were examined at a magnification of 5000× and a scale of 20 μm.

All analyses were performed at room temperature, and the film hydrogel samples were measured at least in triplicate.

### 4.5. Antibacterial Test

The microorganisms evaluated in this study were *Streptococcus mutans* ATCC 25175, *Streptococcus salivarius* ATCC 13419, *Porphyromonas gingivalis* ATCC 33277, *Enterococcus faecalis* ATCC 29212, *Escherichia coli* ATCC 25922, and *Staphylococcus aureus* ATCC 25923, all obtained from the Microbiology Laboratory collection of the Faculty of Biology and Geology, BabeşBolyai University, Cluj-Napoca, Romania.

Each bacterial strain was cultured for 24 h on Nutrient Agar medium [68], after which 0.5 McFarland suspensions were prepared in sterile saline. Using a sterile swab soaked in the 0.5 McFarland suspension, the entire surface of Mueller–Hinton agar plates (Oxoid Ltd., Basingstoke, UK) was inoculated, followed by a 15 min drying at 37 °C.

Wells of 6 mm in diameter were then created in the solid medium, and the prepared hydrogel samples were added. The inoculated plates were kept in the dark for 5 min prior to irradiation. For each bacterial strain, duplicate plates were prepared: one set subjected to irradiation with Laser (LX16 Plus, Woodpecker, Woodpecker.co S.A., Wroclaw, Poland) at 420 nm, and the other kept as a non-irradiated control. Each well was irradiated for 60 s before incubation.

Both irradiated and non-irradiated plates were incubated for 72 h at 25 °C, during which inhibition zones were periodically monitored for each hydrogel and microbial strain. Antimicrobial activity was evaluated by measuring the diameter of the inhibition zones: larger diameters indicated higher bacterial sensitivity to the tested substances [69].

## Figures and Tables

**Table 1:** Composition of the experimental hydrogels tested in the present study.

Hydrogels	Components	GO	GO-HA	GO-ZnO	GO-TiO_2_	GO-SiO_2_	GO-Ag	g-C_3_N_4_	pH
H0	Polyethylene glycol (PEG) 400 Sodium alginate, Whey protein isolate, Glycerine, Distilled water	-	-	-	-	-	-	-	6
H1		0.1%							6
H2			1.5%			-			7
H3		-		0.1%		-			6
H4		-	-		0.1%				6
H5						0.1%			6
H6							0.1%		6
H7								0.1%	7

## Data Availability

The original contributions presented in the study are included in the article; further inquiries can be directed to the corresponding author.
